# Comprehensive analysis revealed the immunoinflammatory targets of rheumatoid arthritis based on intestinal flora, miRNA, transcription factors, and RNA-binding proteins databases, GSEA and GSVA pathway observations, and immunoinfiltration typing

**DOI:** 10.1186/s41065-024-00310-6

**Published:** 2024-01-25

**Authors:** Yin Guan, Yue Zhang, Xiaoqian Zhao, Yue Wang

**Affiliations:** 1 Affiliated Hospital of Nanjing University of Chinese Medicine, Nanjing, 210029 Jiangsu China; 2 Department of Ethics Committee, Affiliated Hospital of Nanjing University of Chinese Medicine, Nanjing, 210029 Jiangsu China; 3 Department of Rheumatism Immunity Branch, Affiliated Hospital of Nanjing University of Chinese Medicine, No. 155 Hanzhong Road, Nanjing, 210029 Jiangsu China

**Keywords:** Rheumatoid arthritis, Biomarkers, Pathogenesis, Bioinformatics analysis

## Abstract

**Objective:**

Rheumatoid arthritis (RA) is a chronic inflammatory arthritis. This study aimed to identify potential biomarkers and possible pathogenesis of RA using various bioinformatics analysis tools.

**Methods:**

The GMrepo database provided a visual representation of the analysis of intestinal flora. We selected the GSE55235 and GSE55457 datasets from the Gene Expression Omnibus database to identify differentially expressed genes (DEGs) separately. With the intersection of these DEGs with the target genes associated with RA found in the GeneCards database, we obtained the DEGs targeted by RA (DERATGs). Subsequently, Disease Ontology, Gene Ontology, and the Kyoto Encyclopedia of Genes and Genomes were used to analyze DERATGs functionally. Gene Set Enrichment Analysis (GSEA) and Gene Set Variation Analysis (GSVA) were performed on the data from the gene expression matrix. Additionally, the protein-protein interaction network, transcription factor (TF)-targets, target-drug, microRNA (miRNA)-mRNA networks, and RNA-binding proteins (RBPs)-DERATGs correlation analyses were built. The CIBERSORT was used to evaluate the inflammatory immune state. The single-sample GSEA (ssGSEA) algorithm and differential analysis of DERATGs were used among the infiltration degree subtypes.

**Results:**

There were some correlations between the abundance of gut flora and the prevalence of RA. A total of 54 DERATGs were identified, mainly related to immune and inflammatory responses and immunodeficiency diseases. Through GSEA and GSVA analysis, we found pathway alterations related to metabolic regulations, autoimmune diseases, and immunodeficiency-related disorders. We obtained 20 hub genes and 2 subnetworks. Additionally, we found that 39 TFs, 174 drugs, 2310 miRNAs, and several RBPs were related to DERATGs. Mast, plasma, and naive B cells differed during immune infiltration. We discovered DERATGs’ differences among subtypes using the ssGSEA algorithm and subtype grouping.

**Conclusions:**

The findings of this study could help with RA diagnosis, prognosis, and targeted molecular treatment.

**Supplementary Information:**

The online version contains supplementary material available at 10.1186/s41065-024-00310-6.

## Introduction

A systemic autoimmune disease called rheumatoid arthritis (RA) is characterized by chronic inflammation that can damage joints and extra-articular organs [1]. It deteriorates intermittently, and without proper therapy, the symptoms worsen over time until the joints are irreparably damaged, leading to additional physical and psychological issues [2]. Therefore, managing and preventing RA requires early identification, diagnosis, and management. Some studies have shown that successful early intervention can significantly lower the financial burden of RA [3, 4]. However, the early onset of RA is typically misleading and challenging to identify at first [5]. Rheumatoid factors, anti-citrullinated protein antibodies (ACPAs), erythrocyte sedimentation rate, and C-reactive protein are the only four biomarkers currently used to identify RA, and each has some limitations [6]. Conventional, biological, and novel abiotic disease-modifying antirheumatic drugs are also recognized treatment options. A composite score is also used to quantify disease activity. While most patients respond to the available treatments and experience remission, many do not or are resistant [7]. Therefore, it is crucial to thoroughly comprehend the evolving mechanism of RA, search for novel signs that might be used for clinical diagnosis or identification of RA conditions, and design more efficient medication treatment targets. Based on the issues mentioned above and their significance, we suggest the following scientific questions: Several mechanisms can be identified and diagnosed in RA, and some genetic characteristics could serve as new targets for clinical treatment with current drugs.

**Fig. 1:**
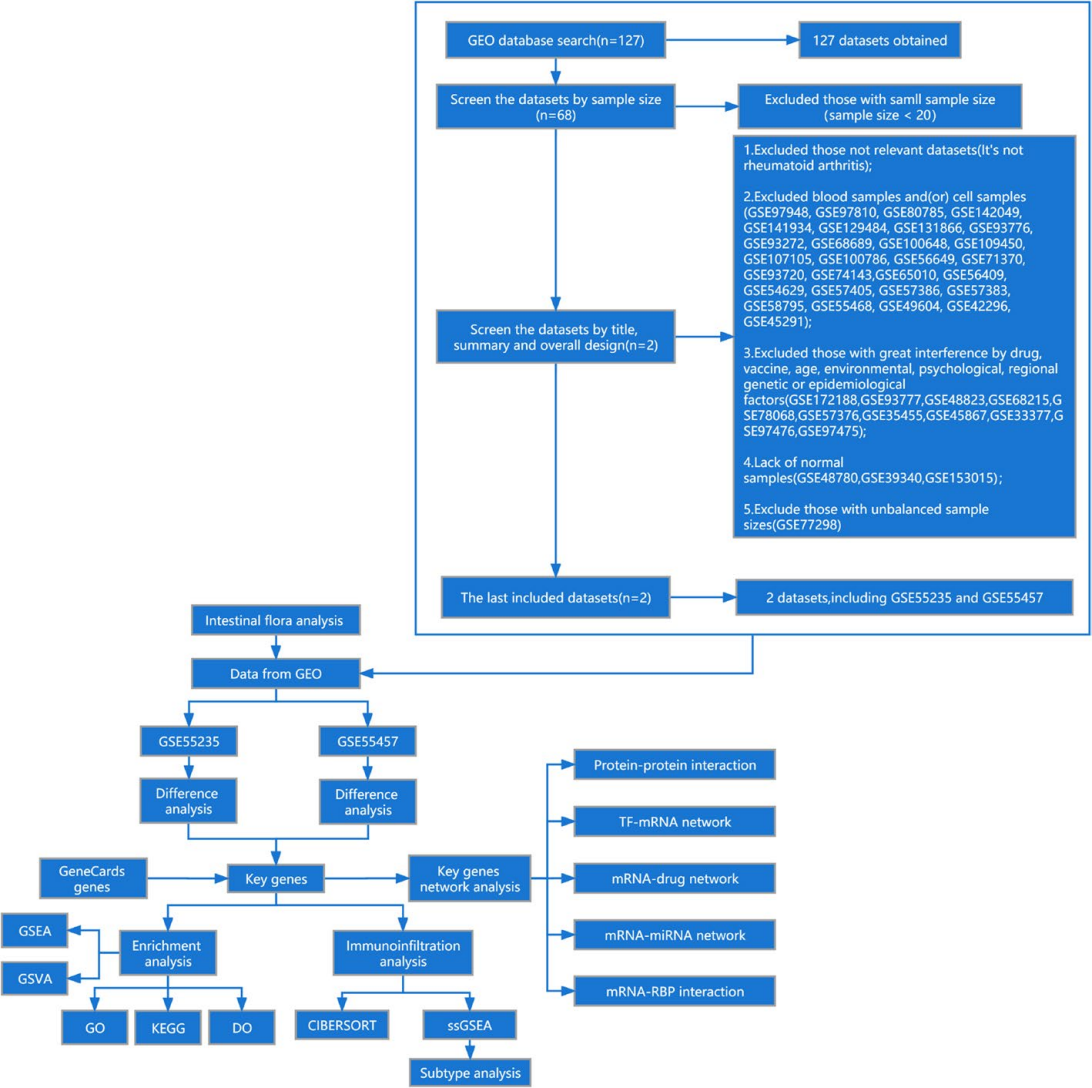
Flowchart of the analytical process

**Fig. 2:**
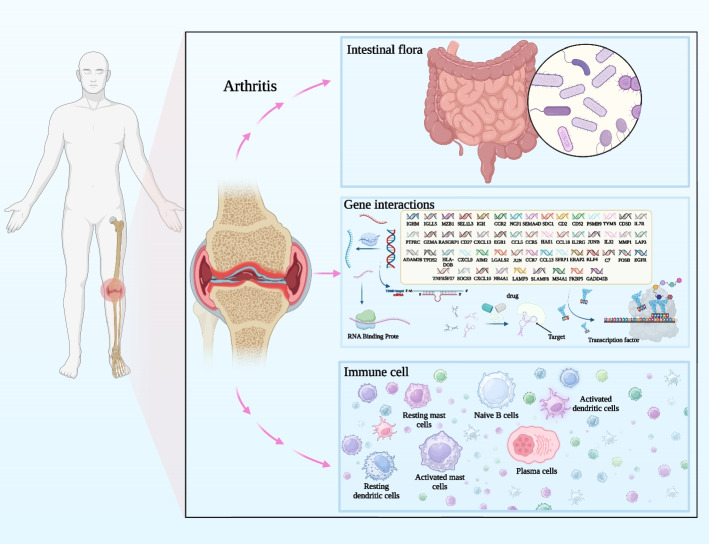
Main discovery mechanism diagram

We used various bioinformatics analytical methods to examine biomarkers and the inflammatory status of RA, including R packages from Bioconductor; the databases Gene Expression Omnibus (GEO), GMrepo, GeneCards, STRING, PharmGKB, DrugCentral, TargetScan, RNA-Binding Protein DataBase (RBPDB); the Cytoscape software; and the CIBERSORT website. This study provided a comprehensive reference for the current RA treatment conundrum by thoroughly explaining every aspect of the pathological molecular mechanism of RA and thoroughly analyzing the druggable targets that can be used for clinical diagnosis and treatment based on the mined key gene targets. Figure 1 depicts the workflow of the current study. Figure 2 summarizes the main findings of this study.

## Materials and methods

### The intestinal flora analysis

The GMrepo database (https://gmrepo.humangut.info/home) was used to retrieve relevant intestinal microbiotas of RA [8]. A correlation map was constructed between the relative abundance of gut microbiota and the prevalence of RA. A species co-occurrence network map was constructed by analyzing the relationships between species or genera of gut microbiota in patients with RA.

### Search strategy for GEO datasets

The following keywords were used to systematically retrieve 127 datasets from the GEO database (https://www.ncbi.nlm.nih.gov/geo/) ((((rheumatoid arthritis[MeSH Terms]) OR rheumatoid arthritis) AND human[Organism]) AND Expression profiling by array[Filter]) AND (“2012/01/01”[Publication Date]: “2022/01/01”[Publication Date]). Exclusion criteria: (1) Excluded those with a small sample size (sample size < 20), (2) Excluded those with irrelevant datasets (no rheumatoid arthritis), (3) Excluded blood samples and/or cell samples, (4) Excluded those with significant interference from the drug, vaccine, age, environmental, psychological, regional genetic, or epidemiological factors, (5) Lack of normal samples, and (6) Excluded those with unbalanced sample sizes (listed in Fig. 1).

### Data download and preprocessing

The GEO database (https://www.ncbi.nlm.nih.gov/geo/) was used to download microarray datasets GSE55235 [9] and GSE55457 [9] using the R package (GEOquery) [10]. Additionally, all dataset samples were generated from *Homo sapiens* using the GPL96 [HG-U133A] Affymetrix Human Genome U133A Array platform. The GSE55235 dataset contained 10 samples from patients with RA and 10 samples from healthy volunteers. In contrast, the GSE55457 dataset contained 13 samples from patients with RA and 10 samples from healthy volunteers, which were used in this study. RMA algorithm from the Affy package in R was used to normalize the data [11]. With the RNASeqSampleSize package, statistical power analysis of the data is done [12].

### Differentially Expressed Genes (DEGs) screening and functional analysis

The DEGs for the two datasets, GSE55235 and GSE55457, were identified using limma [13]. The DEGs were then displayed using the R program as Volcano plots and Heat maps using the ggplot2 and pheatmap packages, respectively. | log2 of the Fold Change (log2FC)|> 1 and adjusted *P*-value < 0.05 were used to recruit DEGs. The GeneCards database (http://www.genecards.org/) was used to find the RA target genes (RATGs) [14] by searching the keyword “rheumatoid arthritis.” Next, the DEGs targeted by RA (DERATGs) were filtered by overlapping the DEGs and RATGs using a Venn diagram. Subsequently, the clusterProfiler package [15] was used to handle Gene Ontology (GO) function, and Kyoto Encyclopedia of Genes and Genomes (KEGG) pathway enrichments on DERATGs and Disease Ontology (DO) enrichments were performed for DERATGs using the DOSE package [16]. Adjusted *P*-value < 0.05 was considered statistically different. Meanwhile, the Gene Set Enrichment Analysis (GSEA) was performed on all RA genes (previously ranked based on their log2FC between analyzed groups) using the clusterProfiler package. It was thought that the enrichment was significant if the nominal false discovery rate (FDR) < 0.25 and *P*-value < 0.05 by referencing the “c2.cp.kegg.v7.5.1.symbols.gmt” gene set. Utilizing the gene set variation analysis (GSVA) package [17], RA gene expression matrix data were subjected to GSVA. The differential pathways were filtered according to adjusted *P*-value < 0.05 and |log2FC|> 0.263.

### Construction of the Protein–Protein Interaction (PPI) network

The PPI network of the DERATGs was analyzed using the interaction relation in the database STRING (https://string-db.org/) [18]. Network node attributes were calculated using NetworkAnalyzer in Cytoscape [19]. Cytoscape’s cytoHubba plugin [20] predicted important nodes (or hub proteins). The subnetworks were extracted from the whole PPI network using the MCODE [20].

### Construction of DERATGs related networks

Transcription factor (TF)-target relationships data was obtained from the TRRUST database (https://www.grnpedia.org/trrust/) [21]. Meanwhile, the Cytoscape software also showed its relational network. The database PharmGKB (https://www.pharmgkb.org/) [22] and the database DrugCentral (https://drugcentral.org/) [23] forecasted the association of interactions between DERATGs and medicinal compounds. The microRNA (miRNA)-mRNA regulatory networks were constructed using the TargetScan database (http://www.targetscan.org/vert_71/) [24] to predict the potentially related miRNA of DERATGs. The database RBPDB (http://rbpdb.ccbr.utoronto.ca/) was used to predict the RNA-binding proteins of DERATGs [25].

### Analysis of immune cell infiltration

CIBERSORT deconvolves the transcriptome expression matrix to determine the make-up and number of immune cells within a mixed cell population using linear support vector regression [26]. We entered the gene expression matrix data into CIBERSORT, filtered samples with a *P*-value < 0.05, and created the immune cell infiltration matrix. The ggheatmap package generated heat maps depicting the 22 immune cells during each sample. Boxplots were created using the ggplot2 and ggpubr packages to investigate differences in immune infiltrating cells between groups, with *P* < 0.05 as the screening standard.

### Subgroup evaluation

Twenty-eight RA samples were given enrichment scores using single-sample GSEA (ssGSEA) [27]. Following that, depending on immune infiltrating cell expression, we categorized RA into two subtypes (Cluster1 and Cluster2). We investigated the DEGs of DERATGs between these subtypes in the GSE55235 and the GSE55457 datasets.

## Results

### Statistical analyses of the intestinal microbiota

**Fig. 3:**
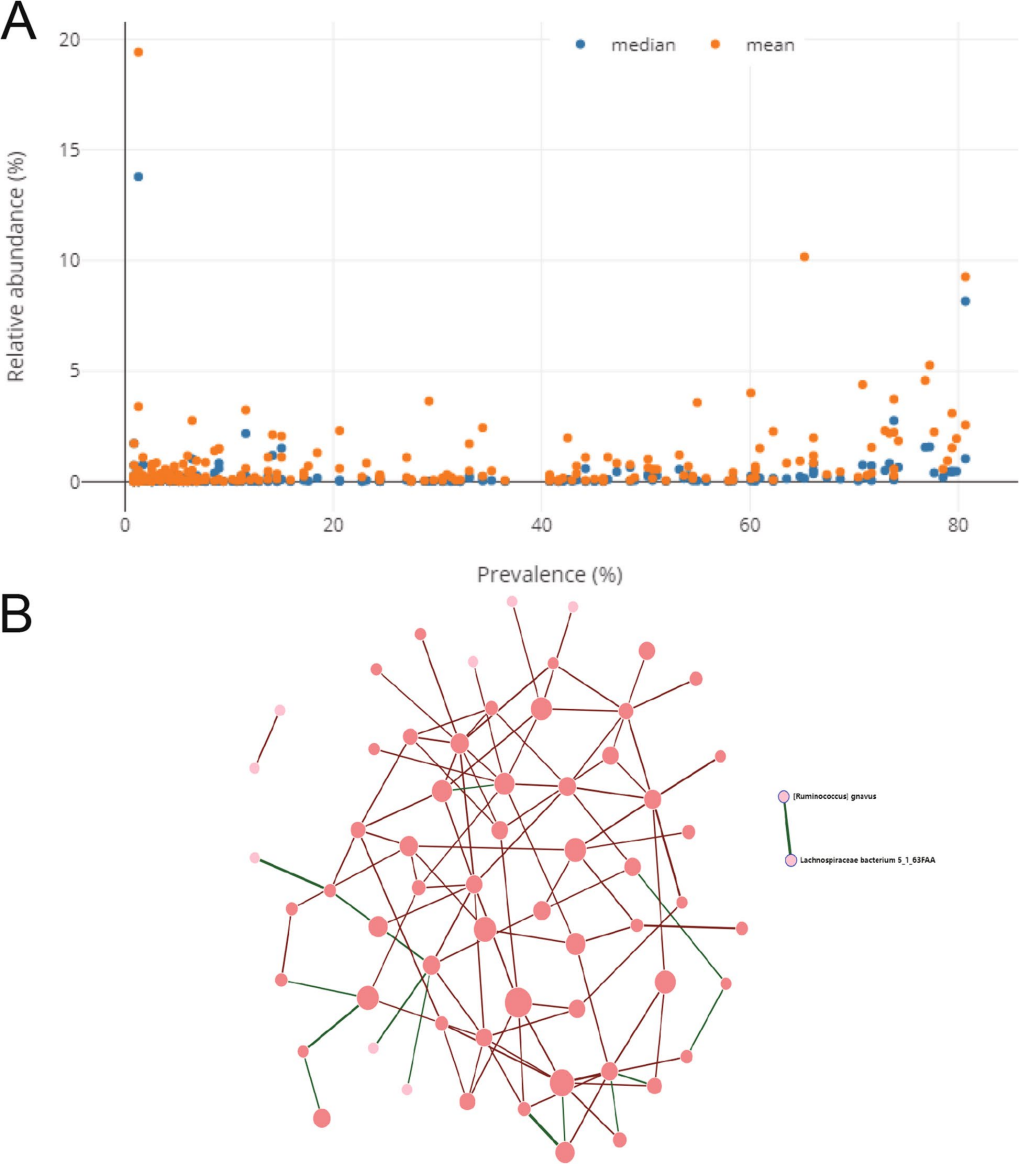
Data on intestinal microbiota in rheumatoid arthritis. **A** Prevalence abundance map analysis. **B** Species co-occurrence network diagram. Green indicates positive, and red indicates negative correlations

A correlation map between the abundance of gut microbiota and the prevalence of RA was created using the GMrepo database (Fig. 3A). The percentage of samples that contained species/genera > 0.01% abundance threshold was counted, and the mean/median relative abundance of species in all RA samples was also summarized. The species co-occurrence network diagram (Fig. 3B) was constructed, with nodes representing species or genera co-occurring with other species or genera in this RA sample. The number of connected network nodes affects the size, and the width of the lines (Pearson’s correlation) represents relationships between species or genera characterized by co-occurrence.

### Data selection and DERATGs screening

**Fig. 4:**
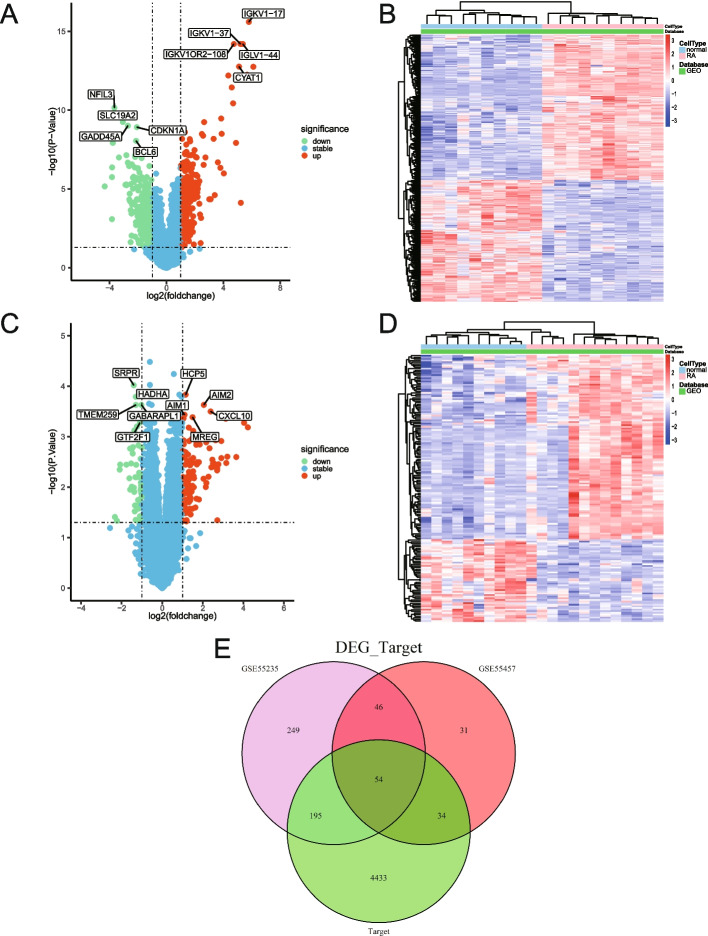
Differentially expressed genes targeted by RA (DERATGs) screening. **A** Volcano plot of GSE55235. Red signifies upregulated DEGs, green signifies downregulated DEGs, and blue signifies no DEGs. **B** Heat map of GSE55235. Blue represents the normal group, and red represents the rheumatoid arthritis (RA) group. **C** Volcano plot of GSE55457. Red indicates upregulated DEGs, green indicates downregulated DEGs, and blue indicates no DEGs. **D** Heat map of GSE55457. Blue represents the normal group, and red represents the RA group. **E** Comprehensive screening of DERATGs by DEGs of the two datasets and GeneCards

According to the GEO data platform, the analysis data were summarized and sorted (Table 1). Following data comparison, it was determined that the sample sizes for the RA and the normal groups were roughly balanced, and an examination of the statistical power of the sample sizes of the two data sets was conducted (Table 1), laying the groundwork for further analysis. The GSE55235 dataset’s gene expression matrix was initially standardized and processed. As shown in the Volcano plot (Fig. 4A) created using R software after data preprocessing, the gene expression matrix yielded 296 upregulated and 248 downregulated genes. The top 10 genes with the most significant differences were identified. Subsequently, we also showed the heat map of DEGs for the GSE55235 dataset (Fig. 4B). Following standardization, the GSE55457 dataset was compared to the normal samples. Differential analysis was conducted to obtain 114 genes that were upregulated and 51 genes that were downregulated. The Volcanic plot was shown (Fig. 4C). Meanwhile, the heat map was also used to show the expression between samples (Fig. 4D). GeneCards retrieved the disease targets of RA (Supplement Spreadsheet S1). Differential genes of the two datasets and the target genes of the GeneCards database intersected, and 54 DERATGs were finally screened out (Fig. 4E).

**Table 1:** Data information summary

GEO accession	Platforms	Sample		Statistical Power
**GSE55235**	**GPL96**	**Normal**	**10**	**0.8466**
		**RA**	**10**	
**GSE55457**	**GPL96**	**Normal**	**10**	**0.9212**
		**RA**	**13**

### GO, KEGG, and DO enrichment analysis

**Fig. 5:**
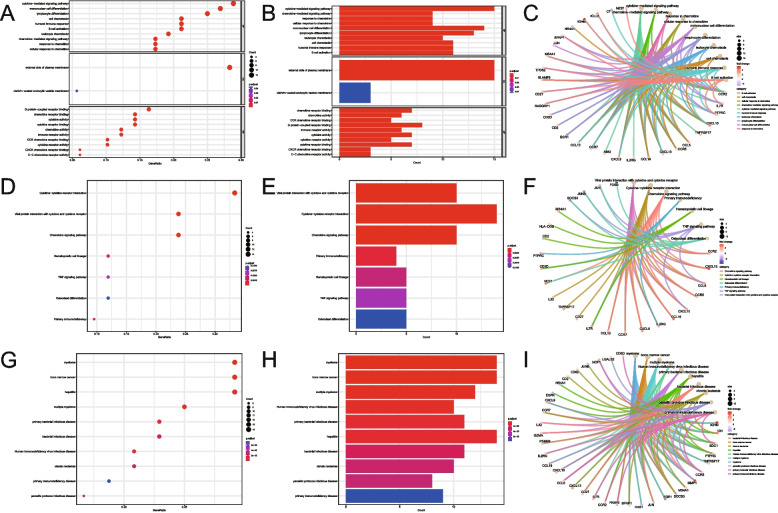
Differentially expressed genes targeted by RA (DERATGs) functional correlation evaluation. **A** DERATGs’ Gene Ontology (GO) biological function enrichment evaluation. The X-axis represents the enrichment of DERATG in GO entries, and the color of the dots represents the adjusted *P*-value: redder is displayed when the adjusted *P*-value is lower, and bluer is displayed when the adjusted *P*-value is higher. The size of the dots serves as a proxy for the number of enriched genes. **B**, **C** Exhibition of DERATGs GO biological function enrichment. **D** DERATGs’ KEGG pathway enrichment study. The size of the dots indicates the number of enriched genes. **E**, **F** DERATGs KEGG biological function enrichment exhibition. **G** DERATGs’ enrichment study using Disease Ontology (DO). The x-axis represents the percentage of DERATGs enriched in the disease team, the y-axis displays the names of the enrichment diseases, and the dot color indicates the adjusted *P*-value: a lower adjusted *P*-value corresponds to red color, and a higher adjusted *P*-value corresponds to blue color. The size of the dots serves as a proxy for the number of enriched genes. **H**, **I** DERATGs DO enrichment exhibition

Then, functional enrichment analyses for GO (Table 2), KEGG (Table 3), and DO (Table 4) were carried out on DERATGs. The GO results confirmed that DERATGs were primarily linked to the cytokine-mediated signaling pathway, clathrin-coated endocytic vesicle membrane, G protein-coupled receptor binding, and other biological phenomena (Fig. 5A-C). The KEGG results showed that the tumor necrosis factor signaling pathway, chemokine signaling pathway, osteoclast differentiation, and other pathways had higher DERATG concentrations than other pathways (Fig. 5D-F). According to DO findings, DERATGs were particularly enriched in myeloma, bone marrow cancer, multiple myeloma, and other diseases (Fig. 5G-I).

**Table 2:** GO enrichment summary

Ontology	ID	Description	*p*.adjust	Count
BP	GO:0019221	cytokine-mediated signaling pathway	< 0.001	15
BP	GO:0070098	chemokine-mediated signaling pathway	< 0.001	9
BP	GO:1990868	response to chemokine	< 0.001	9
BP	GO:1990869	cellular response to chemokine	< 0.001	9
BP	GO:1903131	mononuclear cell differentiation	< 0.001	14
BP	GO:0030098	lymphocyte differentiation	< 0.001	13
BP	GO:0030595	leukocyte chemotaxis	< 0.001	10
BP	GO:0060326	cell chemotaxis	< 0.001	11
BP	GO:0006959	humoral immune response	< 0.001	11
BP	GO:0042113	B cell activation	< 0.001	11
CC	GO:0009897	external side of plasma membrane	< 0.001	15
CC	GO:0030669	clathrin-coated endocytic vesicle membrane	0.043	3
MF	GO:0042379	chemokine receptor binding	< 0.001	7
MF	GO:0008009	chemokine activity	< 0.001	6
MF	GO:0048020	CCR chemokine receptor binding	< 0.001	5
MF	GO:0001664	G protein-coupled receptor binding	< 0.001	8
MF	GO:0140375	immune receptor activity	< 0.001	6
MF	GO:0005125	cytokine activity	< 0.001	7
MF	GO:0004896	cytokine receptor activity	< 0.001	5
MF	GO:0005126	cytokine receptor binding	< 0.001	7
MF	GO:0045236	CXCR chemokine receptor binding	< 0.001	3
MF	GO:0016493	C–C chemokine receptor activity	< 0.001	3

**Table 3:** KEGG enrichment summary

ID	Description	*p*.adjust	qvalue	Count
hsa04061	Viral protein interaction with cytokine and cytokine receptor	< 0.001	< 0.001	10
hsa04060	Cytokine-cytokine receptor interaction	< 0.001	< 0.001	14
hsa04062	Chemokine signaling pathway	< 0.001	< 0.001	10
hsa05340	Primary immunodeficiency	0.001	0.001	4
hsa04640	Hematopoietic cell lineage	0.004	0.003	5
hsa04668	TNF signaling pathway	0.006	0.005	5
hsa04380	Osteoclast differentiation	0.01	0.008	5

**Table 4:** DO enrichment summary

ID	Description	*p*.adjust	qvalue	Count
DOID:0070004	myeloma	< 0.001	< 0.001	14
DOID:4960	bone marrow cancer	< 0.001	< 0.001	14
DOID:9538	multiple myeloma	< 0.001	< 0.001	12
DOID:526	Human immunodeficiency virus infectious disease	< 0.001	< 0.001	10
DOID:0050338	primary bacterial infectious disease	< 0.001	< 0.001	11
DOID:2237	hepatitis	< 0.001	< 0.001	14
DOID:104	bacterial infectious disease	< 0.001	< 0.001	11
DOID:1036	chronic leukemia	< 0.001	< 0.001	10
DOID:2789	parasitic protozoa infectious disease	< 0.001	< 0.001	8
DOID:612	primary immunodeficiency disease	< 0.001	< 0.001	9

### GSEA and GSVA analysis

**Fig. 6:**
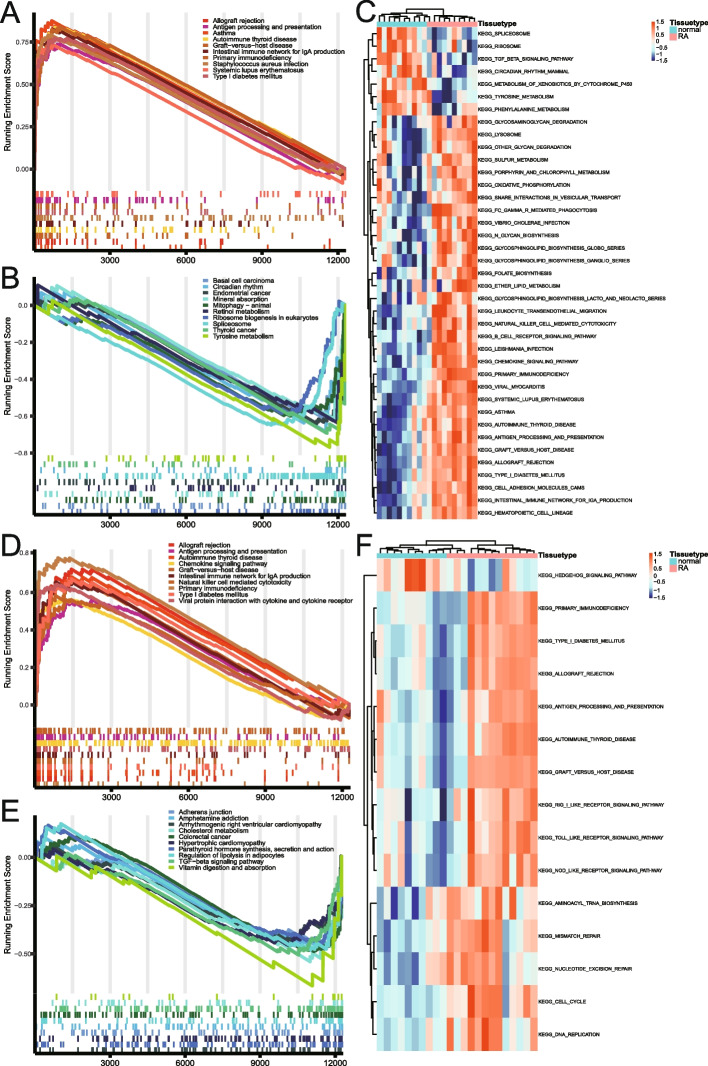
Gene Set Enrichment Analysis and Gene Set Variation Analysis of the GSE55235 and GSE55457 datasets. **A** Analysis of differential genes’ upregulated pathways in the GSE55235 dataset. **B** Analysis of differential genes’ downregulated pathways in the GSE55235 dataset. **C** Differentially enriched pathways in the GSE55235 dataset. **D** Analysis of differential genes’ upregulated pathways in the GSE55457 dataset. **E** Analysis of differential genes’ downregulated pathways in the GSE55457 dataset. **F** Differentially enriched pathways in the GSE55457 dataset

Our reference gene set was “c2.cp.kegg.v7.5.1.symbols.gmt.” The two datasets were subjected to a GSEA enrichment analysis to identify significant enrichment according to the FDR criteria < 0.25 and *P* < 0.05. (Table 5). The GSEA enrichment analysis revealed that the DERATGs in the GSE55235 dataset exist and are also significantly enriched in the upregulated pathways, such as an intestinal immune network for immunoglobulin (Ig)A production, allograft rejection, autoimmune thyroid disease, etc. (Fig. 6A), and are also significantly enriched in the downregulated pathways, such as ribosome biogenesis in eukaryotes, basal cell carcinoma, mitophagy-animal, and so on (Fig. 6B). Similarly, the GSEA enrichment analysis on the GSE55457 dataset (Fig. 6D, E) revealed very high similarity with the GSE55235 dataset, demonstrating the efficacy of DERATGs and enabling further analysis. GSVA enrichment analysis was performed on the GSE55235 and the GSE55457 datasets, and distinct pathways were displayed (Table 6). The differential pathways in the GSE55235 dataset included autoimmune thyroid disease, the intestinal immune network for IgA production, viral myocarditis, and so on (Fig. 6C). Its outcomes matched those of the GSE55457’s GSVA differential pathway (Fig. 6F).

**Table 5:** GSEA enrichment summary

GEO accession	ID	Description	enrichmentScore	*p*value	qvalues
GSE55235	hsa05310	Asthma	0.876	< 0.001	< 0.001
	hsa05330	Allograft rejection	0.842	< 0.001	< 0.001
	hsa05340	Primary immunodeficiency	0.841	< 0.001	< 0.001
	hsa05320	Autoimmune thyroid disease	0.837	< 0.001	< 0.001
	hsa04672	Intestinal immune network for IgA production	0.826	< 0.001	< 0.001
	hsa05150	Staphylococcus aureus infection	0.793	< 0.001	< 0.001
	hsa04940	Type I diabetes mellitus	0.773	< 0.001	< 0.001
	hsa05332	Graft-versus-host disease	0.773	< 0.001	< 0.001
	hsa04612	Antigen processing and presentation	0.739	< 0.001	< 0.001
	hsa05322	Systemic lupus erythematosus	0.736	< 0.001	< 0.001
	hsa03008	Ribosome biogenesis in eukaryotes	-0.591	0.004	0.017
	hsa05217	Basal cell carcinoma	-0.606	0.002	0.01
	hsa04137	Mitophagy—animal	-0.607	< 0.001	0.006
	hsa04978	Mineral absorption	-0.617	0.003	0.013
	hsa00830	Retinol metabolism	-0.635	0.004	0.015
	hsa05213	Endometrial cancer	-0.64	< 0.001	0.003
	hsa03040	Spliceosome	-0.649	< 0.001	< 0.001
	hsa04710	Circadian rhythm	-0.685	0.006	0.022
	hsa05216	Thyroid cancer	-0.693	< 0.001	0.006
	hsa00350	Tyrosine metabolism	-0.769	< 0.001	< 0.001
GSE55457	hsa05340	Primary immunodeficiency	0.785	< 0.001	< 0.001
	hsa05330	Allograft rejection	0.724	< 0.001	< 0.001
	hsa05320	Autoimmune thyroid disease	0.683	< 0.001	< 0.001
	hsa04940	Type I diabetes mellitus	0.672	< 0.001	< 0.001
	hsa05332	Graft-versus-host disease	0.661	< 0.001	0.002
	hsa04672	Intestinal immune network for IgA production	0.657	< 0.001	< 0.001
	hsa04061	Viral protein interaction with cytokine and cytokine receptor	0.643	< 0.001	< 0.001
	hsa04062	Chemokine signaling pathway	0.566	< 0.001	< 0.001
	hsa04612	Antigen processing and presentation	0.563	< 0.001	0.001
	hsa04650	Natural killer cell mediated cytotoxicity	0.562	< 0.001	< 0.001
	hsa05412	Arrhythmogenic right ventricular cardiomyopathy	-0.432	0.007	0.034
	hsa04928	Parathyroid hormone synthesis, secretion and action	-0.444	< 0.001	0.006
	hsa05410	Hypertrophic cardiomyopathy	-0.455	0.001	0.008
	hsa04520	Adherens junction	-0.477	0.001	0.01
	hsa05031	Amphetamine addiction	-0.479	0.004	0.023
	hsa04923	Regulation of lipolysis in adipocytes	-0.481	0.004	0.024
	hsa05210	Colorectal cancer	-0.488	< 0.001	0.002
	hsa04350	TGF-beta signaling pathway	-0.506	< 0.001	0.001
	hsa04979	Cholesterol metabolism	-0.541	< 0.001	0.006
	hsa04977	Vitamin digestion and absorption	-0.666	0.005	0.028

**Table 6:** GSVA enrichment summary

GEO accession	Description	logFC	AveExpr	*P*.Value	adj.*P*.Val
GSE55235	KEGG_PRIMARY_IMMUNODEFICIENCY	0.723	0.004	< 0.001	< 0.001
	KEGG_ALLOGRAFT_REJECTION	0.702	0.016	< 0.001	< 0.001
	KEGG_VIRAL_MYOCARDITIS	0.492	0.011	< 0.001	< 0.001
	KEGG_SYSTEMIC_LUPUS_ERYTHEMATOSUS	0.469	0.007	< 0.001	< 0.001
	KEGG_LEISHMANIA_INFECTION	0.479	-0.001	< 0.001	< 0.001
	KEGG_INTESTINAL_IMMUNE_NETWORK_FOR_IGA_PRODUCTION	0.564	0.008	< 0.001	< 0.001
	KEGG_ANTIGEN_PROCESSING_AND_PRESENTATION	0.508	-0.004	< 0.001	< 0.001
	KEGG_GRAFT_VERSUS_HOST_DISEASE	0.633	0.03	< 0.001	< 0.001
	KEGG_TYPE_I_DIABETES_MELLITUS	0.528	0.014	< 0.001	< 0.001
	KEGG_ASTHMA	0.549	0.032	< 0.001	< 0.001
	KEGG_CELL_ADHESION_MOLECULES_CAMS	0.436	0.021	< 0.001	< 0.001
	KEGG_LYSOSOME	0.488	0.029	< 0.001	< 0.001
	KEGG_CHEMOKINE_SIGNALING_PATHWAY	0.322	0.014	< 0.001	< 0.001
	KEGG_LEUKOCYTE_TRANSENDOTHELIAL_MIGRATION	0.352	0.003	< 0.001	< 0.001
	KEGG_AUTOIMMUNE_THYROID_DISEASE	0.512	0.007	< 0.001	< 0.001
	KEGG_FC_GAMMA_R_MEDIATED_PHAGOCYTOSIS	0.345	0.009	< 0.001	< 0.001
	KEGG_HEMATOPOIETIC_CELL_LINEAGE	0.318	0.012	< 0.001	< 0.001
	KEGG_B_CELL_RECEPTOR_SIGNALING_PATHWAY	0.328	0.008	< 0.001	0.001
	KEGG_GLYCOSPHINGOLIPID_BIOSYNTHESIS_GLOBO_SERIES	0.419	0.004	< 0.001	0.001
	KEGG_NATURAL_KILLER_CELL_MEDIATED_CYTOTOXICITY	0.346	0.003	< 0.001	0.001
	KEGG_N_GLYCAN_BIOSYNTHESIS	0.384	0.012	< 0.001	0.002
	KEGG_PORPHYRIN_AND_CHLOROPHYLL_METABOLISM	0.343	0.028	< 0.001	0.002
	KEGG_FOLATE_BIOSYNTHESIS	0.418	0.006	< 0.001	0.003
	KEGG_VIBRIO_CHOLERAE_INFECTION	0.343	0.006	< 0.001	0.004
	KEGG_GLYCOSPHINGOLIPID_BIOSYNTHESIS_GANGLIO_SERIES	0.443	0.017	< 0.001	0.004
	KEGG_SULFUR_METABOLISM	0.461	0.027	< 0.001	0.005
	KEGG_OTHER_GLYCAN_DEGRADATION	0.45	0.022	0.003	0.017
	KEGG_SNARE_INTERACTIONS_IN_VESICULAR_TRANSPORT	0.29	0.014	0.003	0.017
	KEGG_ETHER_LIPID_METABOLISM	0.276	-0.009	0.004	0.02
	KEGG_GLYCOSAMINOGLYCAN_DEGRADATION	0.307	0.018	0.004	0.02
	KEGG_OXIDATIVE_PHOSPHORYLATION	0.338	-0.003	0.006	0.027
	KEGG_GLYCOSPHINGOLIPID_BIOSYNTHESIS_LACTO_AND_NEOLACTO_SERIES	0.272	0	0.007	0.03
	KEGG_TGF_BETA_SIGNALING_PATHWAY	-0.352	-0.012	< 0.001	< 0.001
	KEGG_SPLICEOSOME	-0.43	-0.035	< 0.001	0.001
	KEGG_METABOLISM_OF_XENOBIOTICS_BY_CYTOCHROME_P450	-0.281	-0.015	< 0.001	0.002
	KEGG_CIRCADIAN_RHYTHM_MAMMAL	-0.487	-0.043	< 0.001	0.003
	KEGG_TYROSINE_METABOLISM	-0.264	0.001	< 0.001	0.005
	KEGG_RIBOSOME	-0.365	-0.031	0.008	0.03
	KEGG_PHENYLALANINE_METABOLISM	-0.278	0.009	0.009	0.033
GSE55457	KEGG_MISMATCH_REPAIR	0.477	0.006	< 0.001	0.005
	KEGG_CELL_CYCLE	0.325	0.003	< 0.001	0.005
	KEGG_DNA_REPLICATION	0.456	-0.011	< 0.001	0.015
	KEGG_RIG_I_LIKE_RECEPTOR_SIGNALING_PATHWAY	0.274	0.023	< 0.001	0.015
	KEGG_TYPE_I_DIABETES_MELLITUS	0.459	0.011	< 0.001	0.015
	KEGG_AMINOACYL_TRNA_BIOSYNTHESIS	0.367	0.011	< 0.001	0.015
	KEGG_AUTOIMMUNE_THYROID_DISEASE	0.427	0.01	< 0.001	0.015
	KEGG_GRAFT_VERSUS_HOST_DISEASE	0.505	0.025	< 0.001	0.015
	KEGG_ALLOGRAFT_REJECTION	0.533	0.018	< 0.001	0.015
	KEGG_PRIMARY_IMMUNODEFICIENCY	0.492	0.002	< 0.001	0.016
	KEGG_TOLL_LIKE_RECEPTOR_SIGNALING_PATHWAY	0.267	0.024	0.002	0.025
	KEGG_NUCLEOTIDE_EXCISION_REPAIR	0.295	0.006	0.002	0.025
	KEGG_ANTIGEN_PROCESSING_AND_PRESENTATION	0.344	0.017	0.003	0.027
	KEGG_NOD_LIKE_RECEPTOR_SIGNALING_PATHWAY	0.311	0.012	0.003	0.027
	KEGG_HEDGEHOG_SIGNALING_PATHWAY	-0.274	-0.014	0.001	0.021

### Analysis of PPI using DERATGs

**Fig. 7:**
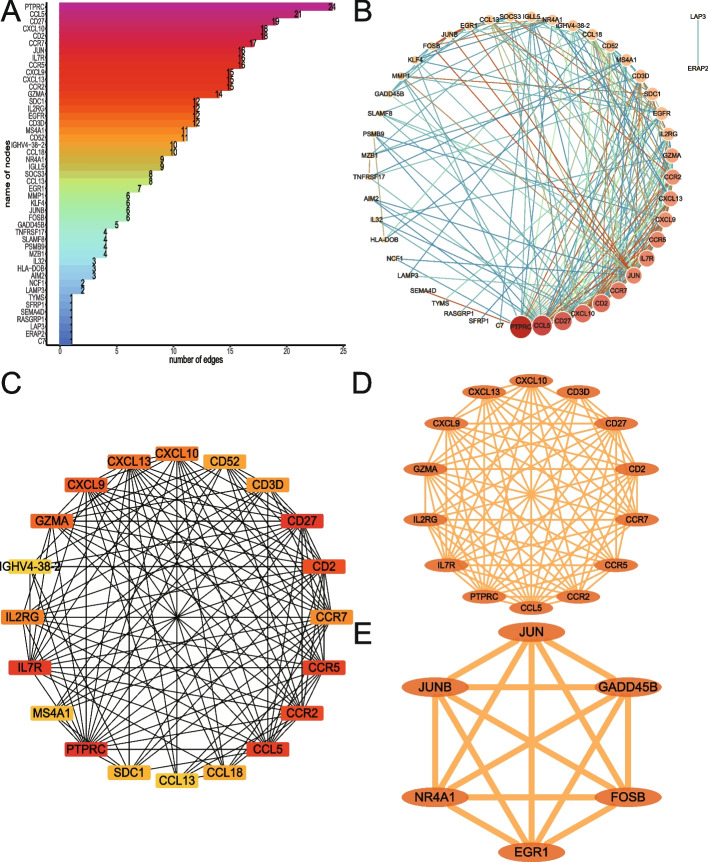
Protein–protein interaction network analysis of differentially expressed genes targeted by RA (DERATGs). **A** Data on the number of protein interaction relationships of DERATGs. **B** Protein interaction network of DERATGs. **C.** Network diagram of the top 20 hub nodes. **D**, **E** Protein interaction network subnetwork construction based on DERATGs, **D** Module 1 and **E** Module 2

PPI of 54 DERATGs was assessed using the database STRING, and 47 DERATGs were discovered to have a PPI link, which was as follows: *C7*, *ERAP2*, *LAP3*, *RASGRP1*, *SEMA4D*, *SFRP1*, *TYMS*, *LAMP3*, *NCF1*, *AIM2*, *HLA-DOB*, *IL32*, *MZB1*, *PSMB9*, *SLAMF8*, *TNFRSF17*, *GADD45B*, *FOSB*, *JUNB*, *KLF4*, *MMP1*, *EGR1*, *CCL13*, *SOCS3*, *IGLL5*, *NR4A1*, *CCL18*, *IGHV4-38-2*, *CD52*, *MS4A1*, *CD3D*, *EGFR*, *IL2RG*, *SDC1*, *GZMA*, *CCR2*, *CXCL13*, *CXCL9*, *CCR5*, *IL7R*, *JUN*, *CCR7*, *CD2*, *CXCL10*, *CD27*, *CCL5*, and *PTPRC*. The number of interactions for each DERATG was visualized (Fig. 7A). Additionally, Cytoscape was used to display the network (Fig. 7B). The node degree increases with an increase in DERATGs size. In contrast, the number of edge interfaces increases as line thickness increases. The cytoHubba tool was used to search hub nodes in the network, and MCC was used to determine the top 20 genes as key gene nodes. The score increases as the node color becomes darker (Fig. 7C). The subnetwork is built using the MCODE, which is used to cluster and build functional modules in the network (Fig. 7D, E), and the construction of the subnetwork reveals the dense areas of potential biological functions.

### Construction of TF-targets, miRNA-mRNA network, and RBP-DERATGs correlation analysis

**Fig. 8:**
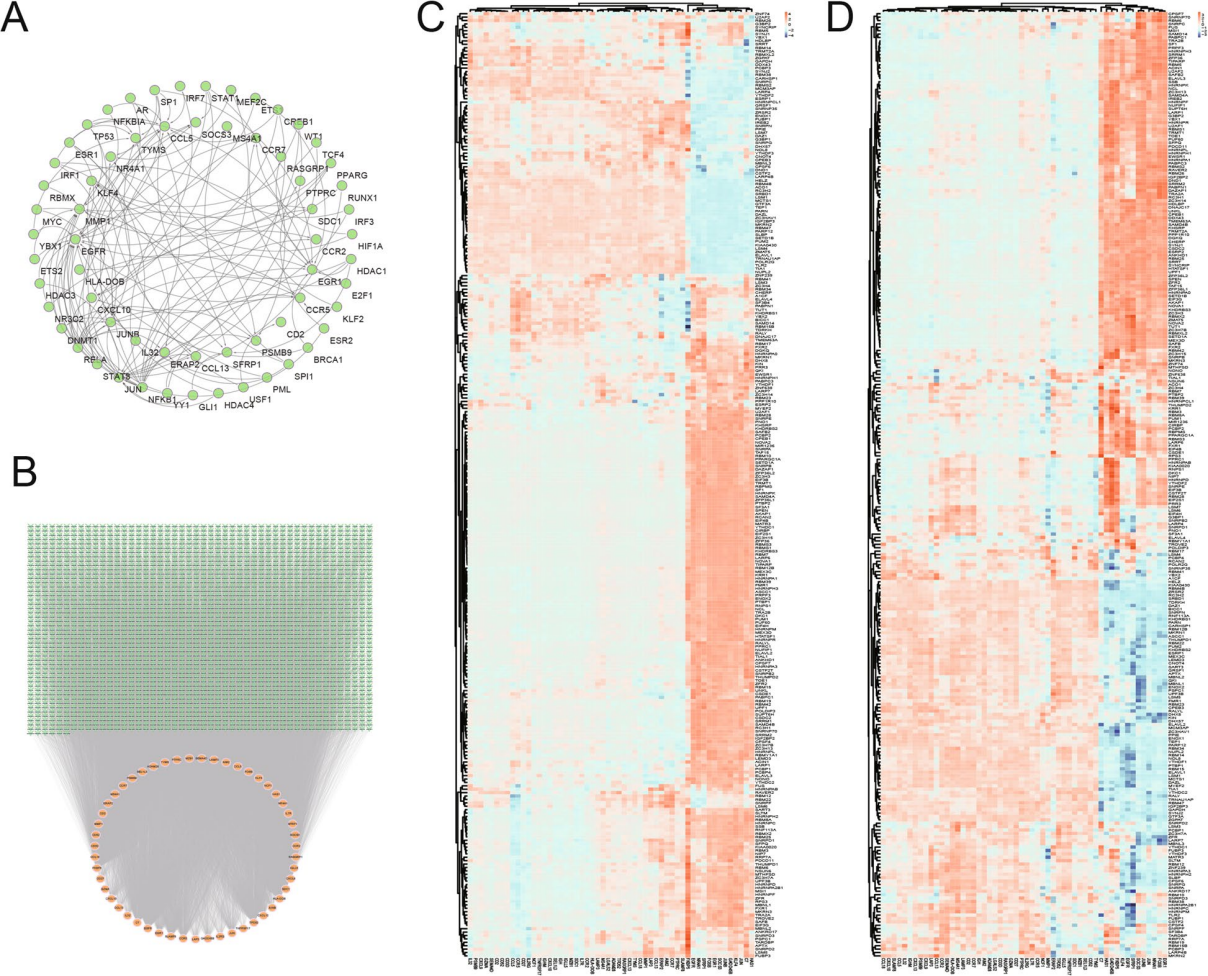
Construction of correlation network and RNA-binding protein (RBP) correlation analysis based on differentially expressed genes targeted by RA (DERATGs). **A** Transcription factor (TF)-target network for DERATGs. The arrow is directed toward the targeted DERATGs from the predicted TF. **B** Micro RNA (miRNA)-mRNA network. Red nodes represent DERATGs, green nodes represent related miRNAs, and lines represent the regulatory relationships between DERATGs and miRNAs. **C** Heat map of the correlation between 54 DERATGs and RBP genes in the expression profile of the GSE55235 dataset. Positively correlated genes are represented by red, whereas negatively correlated genes are represented by blue. **D** Heat map of the correlation between 54 DERATGs and RBP genes in the expression profile of the GSE55457 dataset. Positively correlated genes are represented by red, whereas negatively correlated genes are represented by blue

The TRRUST database predicted the TFs of 54 DERATGs. Thirty-nine TFs were obtained, corresponding to 25 DERATGs. The network visualized the regulatory relationships (Fig. 8A). The TargetScan database also predicted the miRNAs of DERATGs, and 2310 miRNAs were finally predicted to have regulatory relationships with 51 DERATGs. The regulatory relationships were analyzed by network visualization (Fig. 8B). Finally, RBP genes were extracted from the RBPDB database. Correlation analysis was conducted to observe the correlation between RBP genes and 54 DERATGs in the two datasets (GSE55235 and GSE55457) separately, and the results were displayed as heat maps (Fig. 8C, D).

### Construction of interaction networks between drugs and DERATGs targets

**Fig. 9:**
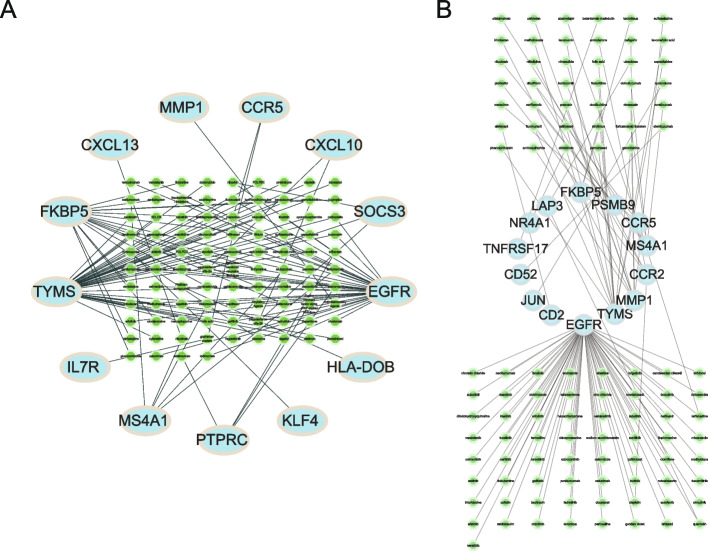
Construction of interaction networks between drugs and DERATGs targets. **A** The construction of a target-drug network via the PharmGKB database. **B** Construction of the target-drug network through the DrugCentral database. Blue indicates DERATGs, and green indicates the drugs predicted by DERATGs

By retrieving the interaction relationship between DERATGs and drugs from the PharmGKB database, finally, we screened these drugs, adalimumab, hydroxyurea, platinum compounds, and so on from the 13 genes analyzed, *PTPRC*, *KLF4*, *HLA-DOB*, *EGFR*, *SOCS3*, *CXCL10*, *CCR5*, *MMP1*, *CXCL13*, *FKBP5*, *TYMS*, *IL7R*, and *MS4A1* (Fig. 9A). Additionally, we looked at the interaction network between DERATGs and targeted drugs through the DrugCentral database, screening for drugs such as ebastine, econazole, ibrutinib, necitumumab, etc., that are associated with *FKBP5*, *PSMB9*, *CCR5*, *MS4A1*, *CCR2*, *MMP1*, *TYMS*, *EGFR*, *CD2*, *JUN*, *CD52*, *TNFRSF17*, *NR4A1*, and *LAP3* (Fig. 9B).

### Immune infiltration analysis

**Fig. 10:**
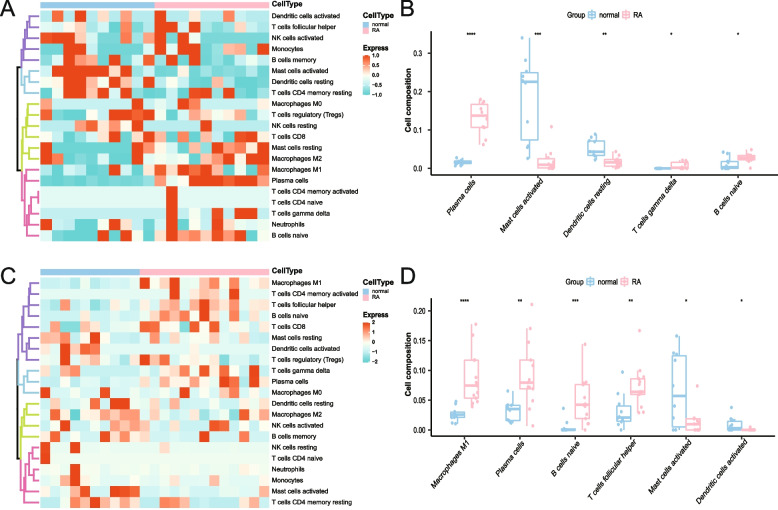
Investigation and visual representation of immune cell infiltration. **A** Heat map of immune infiltration in the GSE55235 dataset shows that rheumatoid arthritis (RA) is indicated by red cells and normal by blue cells. **B** A boxplot depicting significant differential immune infiltration cells in the GSE55235 dataset is colored red for the RA group and blue for the normal group. **C.** Heat map of immune infiltration in the GSE55457 dataset; red cells indicate RA and blue cells indicate normal. **D** A boxplot of the significant differentially immune infiltrating cells of the GSE55457 dataset is shown, with red for the RA group and blue for the normal group. ****p < 0.0001,***p < 0.001, **p < 0.01, and *p < 0.05

The immune cell infiltration of the RA and the normal group samples in the GSE55235 and the GSE55457 datasets were analyzed based on the CIBERSORT algorithm. The immune infiltration of GSE55235 was analyzed, and a heat map was drawn (Fig. 10A). The unexpressed eosinophils cells were eliminated, and only the cells that were expressed in the sample, such as monocytes, follicular helper T cells, neutrophils, etc., were retained in the heat map. The image shows that plasma cells, naive B cells, etc., had a high infiltration level in the RA group, while resting dendritic cells, activated mast cells, etc., had a low infiltration level. Immune cells from different groups were compared (Fig. 10B), and cells with significant differences (*P* < 0.05) are displayed in the figure. Plasma cells, resting dendritic cells, gamma delta T cells, mast cells activated, and naive B cells differed significantly from the heat map. For the GSE55457 dataset, the immune infiltration heat map was also drawn (Fig. 10C). The unexpressed eosinophils cells were eliminated, and only the dendritic cells, monocytes were activated, neutrophils, etc., expressed in the sample were retained in the heat map. However, there was little infiltration of activated mast cells, dendritic cells, and so forth in the RA group. As shown in the figure, the RA group had high levels of infiltration of plasma cells, naive B cells, and others. The differential comparison between groups of the GSE55457 dataset (Fig. 10D) revealed significant differences in the activation of M1 macrophages, plasma cells, naive B cells, follicular helper T cells, activated mast cells, and activated dendritic cells, which was consistent with the results of the GSE55235 dataset.

### Subtype construction based on immune infiltration analysis

**Fig. 11:**
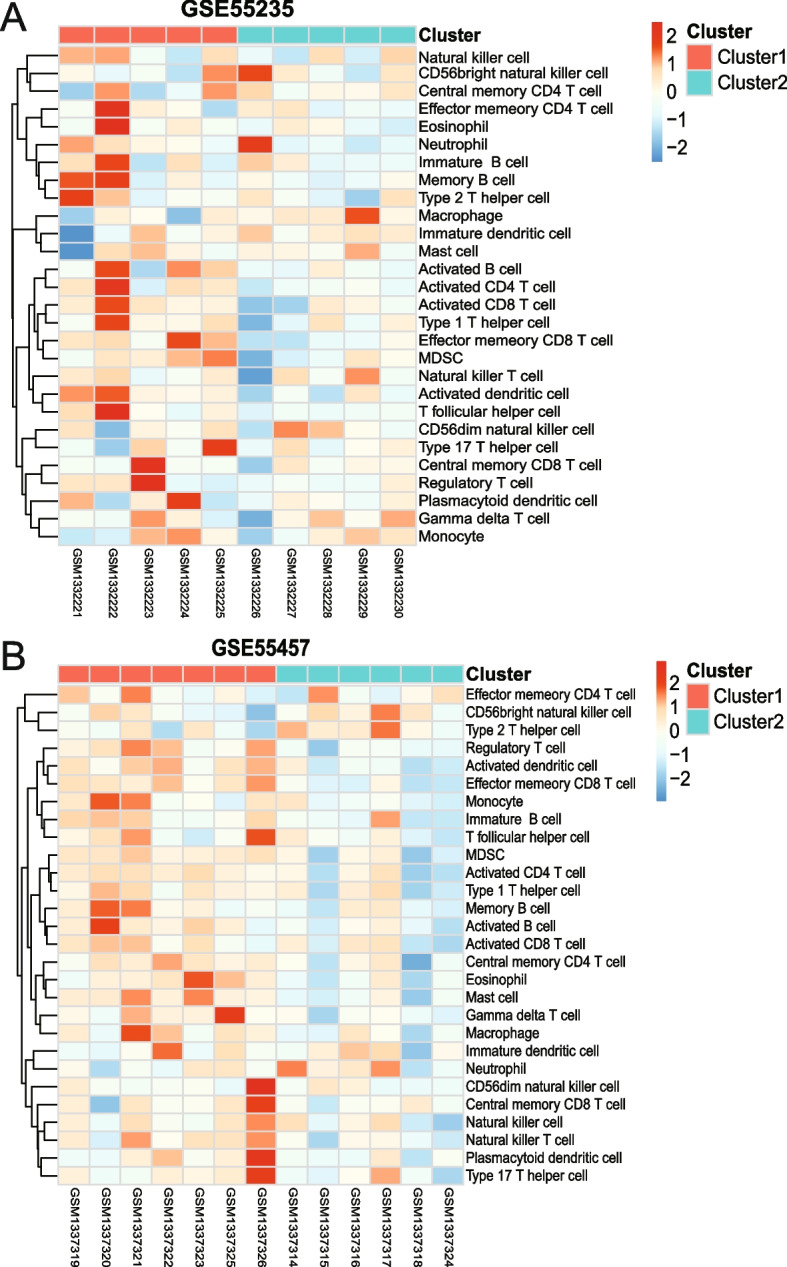
Rheumatoid arthritis sample subtype analysis. **A** Heat map of the 28 distinct types of immune cell infiltration in the GSE55235 dataset. Red and green symbolize the groups in Clusters 1 and 2, respectively. **B** Heat map of immune cell infiltration of 28 different kinds in the GSE55457 dataset. Red and green symbolize the groups in Clusters 1 and 2, respectively

The immune cell infiltration of the RA and normal group samples in the GSE55235 and the GSE55457 datasets was analyzed using the ssGSEA algorithm. Finally, the expression profiles of immune cells were obtained after the expression profiles were predicted and analyzed using 28 types of immune cell-specific marker genes. The RA samples were divided into two subtypes, Cluster1 and Cluster2, by high and low expression clustering (Fig. 11A, B). MDSC, eosinophil, activated CD4 + T cell, and other immune cells were highly expressed in Cluster1 but lowly expressed in Cluster2.

### Differential analysis of DERATGs between subtypes

**Fig. 12:**
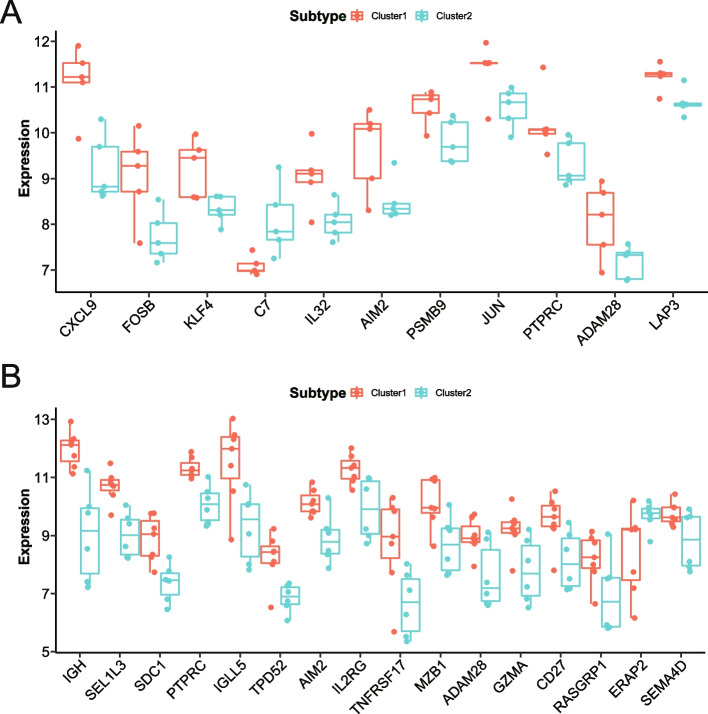
Differential analysis of DERATGs between subtypes. **A** The GSE55235 dataset expresses 11 DERATGs in two subtypes. Red and green symbolize the groups in Clusters 1 and 2, respectively. **B** The GSE55457 dataset expresses 16 DERATGs in two subtypes. Red and green symbolize the groups in Clusters 1 and 2, respectively

The expression of 54 DERATGs in various subtypes was analyzed following the RA subtypes in the GSE55235 and the GSE55457 datasets (Fig. 12A, B). The figure shows the DERATGs that differ significantly (*P* < 0.05). The *C7* gene was significantly expressed in Cluster2 of the GSE55235 dataset, while other significantly different DERATGs were strongly expressed in Cluster1 of the subtype. The *ERAP2* gene was highly expressed in Cluster2 of the GSE55457 dataset, whereas other significantly differential DERATGs were highly expressed in the Cluster1 subtype.

## Discussion

To understand a universal marker assessment is our goal. However, the data sets utilized in this study did not precisely specify factors related to drugs, vaccinations, age, environments, psychology, region, genetics or epidemiology. This does not imply that factors related to these factors did not affect the patients in these two data sets. We removed a few data sets with particular descriptions to prevent bias in this investigation to prevent the proportion of particular subjects from rising.

A total of 54 DERATGs were found by comparing the genes expressed in samples from patients with RA and normal groups. These DERATGs were strongly associated with inflammation and immune response. Although the absence of the well-known three RA star molecules TNF, IL6, and JAK in the DERATGs examined in this study, the KEGG enrichment data showed that DERATGs were enriched in the TNF signaling pathway. In studying biologically targeted drug therapy, these three molecules are often accompanied by biological processes or signaling pathways rather than being studied individually [28–30]. In other words, investigating what seems to be a single molecule is investigating the entire signaling pathway, but these star molecules play an undeniably crucial role in the signaling pathway. Moreover, we discovered several key molecules did not exhibit significant differences in expression changes in actual studies (such as GSEA and GSVA pathway analyses in this study). Therefore, the high setting of the gene screening threshold may be why star molecules were absent from this study.

We performed GSVA and GSEA analyses by analyzing all gene expression data to evaluate further RA’s complex signature of immune/inflammatory responses. Interestingly, the “Intestinal immune network for IgA production” showed high expression in our study, likely supporting our findings on the GMrepo online database. It has been noted that patients with RA (both new-onset and chronic) either showed IgA-like antibody responses to Prevotella copri (P. copri) or its 27-kDa protein, which are associated with the production of TH17 cell cytokines and the presence of ACPAs [31].Additionally, intestinal tissue samples from patients with RA contain higher IgA antibodies that identify dietary antigens [32]. Presently, most RA microbiome studies focus on associations, which aim to link changes in the bacterial composition of the gastrointestinal tract with the condition. Although these findings suggest practical applicability, the mechanism by which gut flora influences the development of RA is still not fully understood [31]. Hence, our study may offer the opportunity to adapt more details and references for future research, diagnostics, and therapeutic approaches.

The inflammatory process in RA depends on chemokines. Multivariate discriminant analysis revealed that chemokines *CXCL10* and *CXCL13* were significantly abundant in the blood plasma of patients with RA compared to healthy volunteers [33]. According to an in vitro study, abatacept’s (ABT) most likely target molecule in inflamed rheumatoid joints is *CXCL10*, and serum *CXCL10* levels may be a feasible predictor of the therapeutic response to ABT treatment [34]. Previous studies have shown that *CCR5* DNA variation impacts the degree of RA severity [35] and that *CCR5* increases the chemotactic response in the synovial fluid of patients with RA [36]. A recent literature review reported that, undoubtedly, *CCR5* had gained its place in RA pathogenesis as an important genetic risk factor [37]. *PTPRC*, also known as *CD45* in some instances, performs several crucial regulatory functions that regulate cell growth, differentiation, mitosis, and malignant transformation [38]. It has been demonstrated to regulate T- and B-cell antigen receptor signaling [39]. In our study, the *PTPRC* gene plays a vital role in this signal transduction network. The PTPRC gene’s roles in RA’s pathogenesis are currently poorly understood.

However, four anti-TNF treatments—TNF-α inhibitors, adalimumab, infliximab, and etanercept—were linked to *PTPRC* in the drug-gene interaction network, demonstrating that *PTPRC* is a druggable gene that can be targeted by TNF-α inhibitors, adalimumab, infliximab, and etanercept. Additionally, *PTPRC* has been demonstrated to be the genetic biomarker of TNFi response most frequently replicated and useful for targeted therapy in patients with RA [40]. Unfortunately, neither IL-6 nor JAK inhibitors showed any evidence of a genetic relationship in our study. This supports the idea that biological processes, rather than specific molecules, are currently the focus of drug research. However, the druggable targets selected for this study offer a broad reference point for future research into new targets for old medicines.

In diseases like cancer, autoimmune disease, diabetes, and cardiovascular disease, TFs play a crucial biological role [41]. Although most TFs have traditionally been regarded as “undruggable” targets [41], current research has revealed that the tumor therapy drug Binimetinib may have a potential targeted binding impact with NFKB1 [42]. Additionally, it has been reported that small molecule inhibitors can specifically target AR, making it the primary treatment target for advanced cancer [43]. This is also something that research has found. Combining ketoprofen and indolamide inhibits the Gli1-mediated transcription in the Hedgehog pathway [44]. It has been demonstrated that the novel oral active molecular gel WBC100 selectively degrades the protein c-Myc over other proteins and effectively kills cancer cells that overexpress c-Myc [45]. Human triple-negative breast and gastric cancer xenografts have been demonstrated to regress in response to WZ-2–033, a new STAT3 inhibitor [46]. According to a study, a bromine domain and extra terminal domain inhibitor can induce tumor cell apoptosis by disrupting the specific transcription network that the TCF4 TF regulates [47]. However, other TFs in this study have not consistently been reported to be pharmacologically actionable. In conclusion, although the majority of present work on druggable TFs focuses on cancer drug development, it also offers suggestions for work on RA-related druggable TFs. We believe that NFKB1, AR, GLI1, Myc, STAT3, and TCF4 are now the most potentially druggable TFs based on the regulatory relationship between TFs and DERATGs in this study.

Currently, cell-type deconvolution analysis is frequently applied in RA research. *FAS*, *MAPK8*, and *TNFSF10* may be associated with alterations in the immune microenvironment in patients with RA, according to a study that used CIBERSORT analysis [48]. It was discovered that *SLC2A3* is positively associated with the expression of activated mast cells in RA synovial tissue using immune cell infiltration [49]. The CIBERSORT study found that the RA key genes *CXCL8*, *CXCL2*, and *FADD* were associated with mast cells, monocytes, activated natural killer cells, CD8 T cells, dormant dendritic cells, and plasma cells [50]. In this study, we performed a thorough analysis of the immune infiltration landscape using ssGSEA and CIBERSORT algorithms to quantify the profile of immune infiltration in RA. Studies have shown that 20% of the antibodies mature naive B cells produce when they reach the periphery are still autoreactive. This percentage is significantly higher in patients with RA [51, 52]. Rituximab, a therapeutic antibody that targets CD20, has been successfully used as a B cell therapy to treat RA. Over the last decade, additional RA studies have suggested that (autoreactive) B cells may contribute to the progression of the disease [53]. There has been no research on RA therapy targeting plasma cells, and the function of plasma cells in RA is still unknown [54].

Regarding the involvement of mast cells and dendritic cells (DC cells) in the pathogenesis of RA, conflicting results have also been found. Although most research has focused on the role of mast cells in the pathogenesis of RA [55–60] and that immature and activated DC cell populations are present in the synovium of the inflamed joint [61]. We found that patients with RA had decreased mast cell and DC cell infiltration in their tissues. However, other studies have suggested that steroid use may be related to decreased mast cells and DC cells in patients with RA [62–64]. The specific cause of the decline in mast cells and DC cells in RA must be further investigated because it is unknown if the patients in this study were using corticosteroids or other drugs.

Cluster1 and Cluster2, subtypes of expression profiles, were identified based on immune cell expression. The purpose of the analysis was to provide a better understanding of the function and regulatory mechanisms of the immune system. We can better understand the function and regulatory mechanisms of immune cells by understanding the expression forms of each subtype through the analysis of gene expression in subtypes. Determine which subtypes share the most common characteristics to determine the most effective course of action. Additionally, subtype expression profiles can also aid in the discovery of novel therapeutic targets. We might identify potential genes or molecules to be used as therapeutic targets by analyzing the genes expressed in specific subtypes. However, further experimental verification is required. The findings indicate that the *PTPRC* gene was highly expressed in Cluster1 in the GSE55235 and GSE55457 datasets. *PTPRC* may be the characteristic gene of the Cluster1 subtype in these two datasets or play a significant biological function that may be directly associated with the function of this subtype. We also noticed discrepancies in the analysis results between the two data sets, which we believe may be due to a batch effect brought on by the differences in data set sample collection location, time and computer sequencing time. Another major limitation of this study is the batch effect.

This study has some other drawbacks. First, the study lacked clinically important details about the condition, such as disease activity and treatment usage. Additionally, no multi-group trials were conducted, and the study’s sole focus was on the gene transcriptome. Finally, bioinformatics approaches limited data analysis; preclinical and clinical validation is required.

In conclusion, the scientific community needs to investigate and comprehend how gut microbiota, genetics, and immune inflammation are related to the etiology of RA. The findings of this study might be used as a reference for clinical diagnosis, prognosis, and targeted molecular treatment for RA.

### Supplementary Information

**Additional file 1.**

## Data Availability

The datasets GSE55235 and GSE55457 for this study can be found in the Gene Expression Omnibus database [https://www.ncbi.nlm.nih.gov/geo/query/acc.cgi?acc=GSE55235/GSE55457]; All the R code and supporting data for this article are available on the GitHub page created by the first author [https://github.com/DrGuanYin686993/The-doctor-is-fond-of-milk.git].

## Appendix

**Table 7:** Gene symbol

Gene.Symbol	Official.Full.Name
**IGHM**	**immunoglobulin heavy constant mu**
**IGLL5**	**immunoglobulin lambda like polypeptide 5**
**MZB1**	**marginal zone B and B1 cell specific protein**
**SEL1L3**	**SEL1L family member 3**
**IGH**	**immunoglobulin heavy locus**
**CCR2**	**C–C motif chemokine receptor 2**
**NCF1**	**neutrophil cytosolic factor 1**
**SEMA4D**	**semaphorin 4D**
**SDC1**	**syndecan 1**
**CD2**	**CD2 molecule**
**CD52**	**CD52 molecule**
**PSMB9**	**proteasome 20S subunit beta 9**
**TYMS**	**thymidylate synthetase**
**CD3D**	**CD3 delta subunit of T-cell receptor complex**
**IL7R**	**interleukin 7 receptor**
**PTPRC**	**protein tyrosine phosphatase receptor type C**
**GZMA**	**granzyme A**
**RASGRP1**	**RAS guanyl releasing protein 1**
**CD27**	**CD27 molecule**
**CXCL13**	**C-X-C motif chemokine ligand 13**
**TNFRSF17**	**TNF receptor superfamily member 17**
**CCL5**	**C–C motif chemokine ligand 5**
**CCR5**	**C–C motif chemokine receptor 5**
**CXCL10**	**C-X-C motif chemokine ligand 10**
**CCL18**	**C–C motif chemokine ligand 18**
**IL2RG**	**interleukin 2 receptor subunit gamma**
**SLAMF8**	**SLAM family member 8**
**HLA-DOB**	**major histocompatibility complex, class II, DO beta**
**MMP1**	**matrix metallopeptidase 1**
**LAP3**	**leucine aminopeptidase 3**
**ADAM28**	**ADAM metallopeptidase domain 28**
**TPD52**	**tumor protein D52**
**IL32**	**interleukin 32**
**CXCL9**	**C-X-C motif chemokine ligand 9**
**AIM2**	**absent in melanoma 2**
**LAMP3**	**lysosomal associated membrane protein 3**
**LGALS2**	**galectin 2**
**CCR7**	**C–C motif chemokine receptor 7**
**CCL13**	**C–C motif chemokine ligand 13**
**MS4A1**	**membrane spanning 4-domains A1**
**ERAP2**	**endoplasmic reticulum aminopeptidase 2**
**KLF4**	**KLF transcription factor 4**
**GADD45B**	**growth arrest and DNA damage inducible beta**
**FOSB**	**FosB proto-oncogene, AP-1 transcription factor subunit**
**EGFR**	**epidermal growth factor receptor**
**SOCS3**	**suppressor of cytokine signaling 3**
**EGR1**	**early growth response 1**
**JUNB**	**JunB proto-oncogene, AP-1 transcription factor subunit**
**C7**	**complement C7**
**NR4A1**	**nuclear receptor subfamily 4 group A member 1**
**JUN**	**Jun proto-oncogene, AP-1 transcription factor subunit**
**HAS1**	**hyaluronan synthase 1**
**SFRP1**	**secreted frizzled related protein 1**
**FKBP5**	**FKBP prolyl isomerase 5**
**TNF**	**tumor necrosis factor**
**IL6**	**interleukin 6**
**JAK1**	**Janus kinase 1**
**JAK2**	**Janus kinase 2**
**JAK3**	**Janus kinase 3**
**TYK2**	**tyrosine kinase 2**
**NFKB1**	**nuclear factor kappa B subunit 1**
**AR**	**androgen receptor**
**GLI1**	**GLI family zinc finger 1**
**MYC**	**MYC proto-oncogene, bHLH transcription factor**
**STAT3**	**signal transducer and activator of transcription 3**
**TCF4**	**transcription factor 4**
**FAS**	**Fas cell surface death receptor**
**MAPK8**	**mitogen-activated protein kinase 8**
**TNFSF10**	**TNF superfamily member 10**
**SLC2A3**	**solute carrier family 2 member 3**
**CXCL8**	**C-X-C motif chemokine ligand 8**
**CXCL2**	**C-X-C motif chemokine ligand 2**
**FADD**	**Fas associated via death domain**
